# Characteristics of outpatient emergency department visits of nursing home residents: an analysis of discharge letters

**DOI:** 10.1007/s40520-021-01863-6

**Published:** 2021-05-03

**Authors:** Stephanie Heinold, Alexander Maximilian Fassmer, Guido Schmiemann, Falk Hoffmann

**Affiliations:** 1 Division of Outpatient Care and Pharmacoepidemiology, Department of Health Services Research, Carl Von Ossietzky University of Oldenburg, Ammerländer Heerstr. 114–118, 26129 Oldenburg, Germany; 2 Institute for Public Health and Nursing Research, University of Bremen, Bremen, Germany; 3 Department of Health Services Research, University of Bremen, Bremen, Germany; 4 Institute for General Practice, Hannover Medical School, Hannover, Germany

**Keywords:** Nursing home residents, Hospital transfers, Outpatient, Treatment, Germany

## Abstract

**Background:**

Unplanned emergency department (ED) visits of nursing home residents (NHR) are common, with many transfers not leading to hospitalization. However, there is little research on what diagnostic and therapeutic measures are performed during visits.

**Aims:**

We analyzed underlying diagnoses, characteristics and performed medical procedures of unplanned outpatient ED visits by NHR.

**Methods:**

We conducted a multi-center study of 14 nursing homes (NHs) in northwestern Germany in 03/2018–07/2019. Hospital transfers were documented by nursing staff using a standardized questionnaire for 12 months. In addition, discharge letters were used to collect information about the respective transfer, its reasons and the extend of the medical services performed in the ED.

**Results:**

A total of 161 unplanned ED visits were included (mean age: 84.2 years; 68.3% females). The main transfer reasons were trauma (59.0%), urinary catheter and nutritional probe problems (overall 10.6%; male NHR 25.5%) and altered mental state (9.9%). 32.9% where discharged without imaging or blood test prior. 67.4% of injured NHR (*n* = 95) required no or only basic wound care. Catheter-related problems (*n* = 17) were mainly treated by changing an existing suprapubic catheter (35.3%) and by flushing the pre-existing catheter (29.4%).

**Discussion:**

Our data suggest that the diagnostic and therapeutic interventions performed in ED, often do not exceed general practitioner (GP) care and many ED visits seem to be unnecessary.

**Conclusion:**

Better coordination and consultation with GPs as well as better training of nursing staff in handling catheter problems could help to reduce the number of ED visits.

## Introduction

The prevalence of emergency department (ED) visits rises with age and is particularly high among nursing home residents (NHR). These patients are a vulnerable, frail and multimorbid group, who suffer from numerous medical comorbidities leading to increased medical complexity [1, 2]. It is often argued that due to limited access to general practitioners (GPs) and specialists, NHR are often transferred to EDs if their condition deteriorates [3]. However, transfers can be burdensome to NHR, because it puts them at risk for in-hospital complications such as pressure ulcers, delirium, iatrogenic infections, and increased mortality [4–6]. Especially those ED visits which do not lead to hospitalization are described as avoidable or inappropriate by many authors [7–9].

Overall, about half of all ED visits by NHR do not lead to hospital admission [10]. This is especially remarkable considering that NHR are more than twice as likely as community dwelling older adults to be admitted to the hospital, once presenting in the ED [11]. The most common reasons for such transfers are trauma (often fall-related), altered mental state and infections [8] and we recently found that 75.0% of NHR not subsequently hospitalized were diagnosed with traumatic injuries [12]. However, little is known about the underlying pathways that led to the transfer decision and the extent of treatment in ED in those not being hospitalized. Recent data suggests that in two-thirds of the transfers the GP was not involved in the decision making [13]. Burke et al. found that 19.0% of the NHR discharged from the ED did not have any diagnostic testing prior [11].

Hence the purpose of this study was to gather information from discharge letters to explore the underlying diagnoses and characteristics of unplanned outpatient ED visits of NHR and the extent of medical services performed.

## Materials and methods

### Study design

This study is part of the HOspitalisations and eMERgency department visits of Nursing home residents (HOMERN) project [14]. In brief, we conducted a multi-center study, including a convenience sample of 14 NHs in northwestern Germany with a total of 802 residents. The included facilities offer full inpatient care by trained nurses and care assistants. As usual in Germany, a GP is not permanently employed by the NHs and, therefore, only available on request. Regarding their location (urban and rural), size (number of beds) and sponsorship (non-profit and private for-profit), the facilities were heterogeneous. Specialized care facilities (e.g., for dementia residents only) and care units that offer short-term care only were excluded. Data was collected between March 2018 and July 2019 for 12 months in each NH. For each hospital transfer (ED visits and hospital admissions, scheduled and unplanned), nursing staff assessed information via questionnaires and were asked to include the related and anonymised discharge letter. There was no need for active participation and informed consent of the residents, because the study relied entirely on information from current medical records and the perceptions of the NH staff. The study was approved by the ethics committee of the Medical Association in Bremen (RA/RE-613, 16 February 2018).

### Data collection and assessed variables

A paper-based, four-page questionnaire developed by a multidisciplinary team was used to document data for each hospital transfer. From that, residents sociodemographic and health characteristics, e.g., dementia diagnosis (yes/no) and stage (three groups), and performance in daily activities of daily living using the modified Barthel Index (BI) developed by Shah et al. [15], were assessed. We used the surprise question (“Would you be surprised if this resident died within the next 6 months?”) to identify NHR reaching the end of life [16]. Moreover, the NH staff was asked if the resident was carrying an advance directive (AD) and stated the details by adjusting the emergency advance directive called the patient directive for life sustaining measures (PALMA) [17]. Further information about the decision-making process (final decision maker, previous contact with other health care specialists) and the respective transfer (date and time slot) were gathered and have been published in detail elsewhere [13].

In addition, the discharge letters were used to collect information about: (a) the transfer and its reasons, (b) the extent of treatment and use of medical resources in the ED, (c) recommendations for further care, and (d) the quality of medical documentation. Regarding the transfer, we recorded the admission mode (walk-in, ambulance, ambulance with prehospital emergency physician). Reasons for ED visit (presenting chief complaints) consisted of the most common reasons for ED transfer according to literature recently found in a systemic review by Lemoyne et al. [8].

Additional notes were made on whether the trauma was fall related. Diagnoses were recorded according to the German modification of the International Classification of Diseases, 10th Revision (ICD-10-GM), whereby only the first two digits were coded (e.g., “Open wound of the eyebrow” = S01). Concerning treatment and resources used, we evaluated the type of diagnostic testing in the ED, including physical examination, blood and urine testing, electrocardiography (ECG), and imaging tests. We collected information about procedures performed in the ED which were categorized in drug administration (e.g., intravenous hydration, sedatives, opioid and non-opioid analgesics), wound care (two groups: ‘basic’ including cooling, wound cleansing, dressing, tetanus vaccination, fibrin glue and wound closure strips and ‘advanced’ including casting and suturing) and treatment of catheter and probe associated problems. Moreover, the indication for catheter/probe treatment was recorded (change of existing catheter/probe or initial installation). Furthermore, we assessed whether and which recommendations for further outpatient care after ED visit were available. Each variable could be rated as ‘not assessable’ if no corresponding data was found in the discharge letters or the questionnaires, which were handled as missing values.

Furthermore, we evaluated the quality of medical documentation using a completeness score, based on the recommendations of the implementation guide for discharge letters used by the German Association of IT Manufacturers for the Healthcare Sector (VHitG) [18]. The score consisted of the five items current anamnesis, essential findings, diagnosis, therapy and further procedure, which are described by the VHitG as standard requirements for discharge letters. Points were awarded for each item so that a maximum score of five could be achieved. Any result below five points was considered incomplete. In addition, structural characteristics of the letters were recorded, such as the length of the documents (based on a DIN A4 page) and whether they were machine or handwritten.

### Statistical analyses

We restricted our analysis to unplanned ED visits by NHR which did not lead to hospitalization and for which a discharge letters were available. Analyses were performed using descriptive statistics. Data were presented as frequency and percentage, and for continuous variables we stated the mean with standard deviation (SD). Because of missing values, denominators differed between variables. We categorized the resident’s age at time of transfer into four groups (≤ 69, 70–79, 80–89 and ≥ 90 years). The Barthel Index (BI) was grouped into five categories according to ICD-10-GM (slight/no dependency; mild dependency; moderate dependency; severe dependency; total dependency). Data was entered into IBM SPSS Statistics for Windows, Version 25.0 (Armonk, NY: IBM Corp.) for statistical analysis.

## Results

### Characteristics of unplanned transferred nursing home residents

A total of 240 ED transfers occurred, which did not lead to hospitalisation. 68 questionnaires were returned without a discharge letter and, therefore, excluded. On closer inspection of the discharge letters, eleven transports turned out to be planned visits for X-ray and pacemaker checks and dialysis appointments. These events were also excluded. The final analysis included 161 ED transfers, ranging from 1 to 28 per NH.

NHR were on average 84.2 years (SD: 9.2) and considerably more often female (68.3%) (see Table 1). Only 10.7% presented with slight/no dependency regarding BI. More than half (59.4%) of the NHR had a dementia diagnosis (81.1% of them in a moderate or severe stage). The nursing staff assessed 32.1% of the NHR as palliative cases with a significantly reduced life expectancy (surprise question, estimating 6-month mortality); however, the resident’s wish for end-of-life care was unknown in 54.0%. From the available ADs 21.1% wanted to be treated exclusively in the NH and not be transferred to hospital.

**Table 1:** Characteristics of unplanned transferred nursing home residents

	Total transfers (*n* = 161)		Transfers of males (*n* = 51)		Transfers of females (*n* = 110)	
**Age of the residents at the time of hospital transfer (years)**^a^						
Mean (SD)	84.2	(9.2)	84.0	(10.6)	84.3	(8.5)
≤ 69	12	7.5%	6	11.8%	6	5.5%
70–79	26	16.3%	9	17.6%	17	15.6%
80–89	82	51.3%	19	37.3%	63	57.8%
≥ 90	40	25.0%	17	33.3%	23	21.0%
**Barthel Index: residents’ activities of daily living (points)**^a^						
Mean (SD)	45.7	(25.5)	41.7	(26.9)	47.6	(24.7)
80–100: slight/no dependency	17	10.7%	5	9.8%	12	11.1%
60–75: mild dependency	42	26.4%	10	19.6%	32	29.6%
40–55: moderate dependency	43	27.0%	14	27.5%	29	26.9%
20–35: severe dependency	25	15.7%	10	19.6%	15	13.9%
0–15: total dependency	32	20.1%	12	25.5%	20	18.5%
**Dementia diagnosis of the residents**^a^						
No	65	40.6%	26	52.0%	39	35.5%
Yes	95	59.4%	24	48.0%	71	64.5%
Stage: mild	17	18.9%	3	13.0%	14	20.9%
Stage: moderate	42	46.7%	10	43.5%	32	47.8%
Stage: severe	31	34.4%	10	43.5%	21	31.3%
**Resident’s wish for end-of-life care**^a^						
Unknown	87	54.0%	30	58.8%	57	51.8%
Advance directive available	74	46.0%	21	41.2%	53	48.2%
Full clinical emergency treatment	2	2.8%	0	0.0%	2	3.9%
Limited clinical treatment	48	67.6%	12	60.0%	36	70.6%
Preclinical emergency treatment in the NH	15	21.1%	8	40.0%	7	13.7%
Assessment not possible	6	8.5%	0	0.0%	6	11.8%
**Surprise question (estimating 6-month mortality)**^a^						
Likely	51	32.1%	20	39.2%	31	28.7%
Unlikely	108	67.9%	31	60.8%	77	71.3%

*SD* standard deviation, *NH* nursing home

^a^Numbers differ due to missing values

The age distribution between men and women was similar. Some characteristics differed between the sexes. On the one hand, women had a higher BI than men. On the other hand, female NHR were more often diagnosed with dementia than men (64.5 vs. 48.0%), while men were more frequently in a severe stage.

### Characteristics of emergency department visits

The ED visits were relatively constant over the weekdays (11.3–18.8%). They most often occurred during office hours from 07:00 to 18:00 h (62.5%). According to the NH staff, about 70% of residents had an acute onset of symptoms with less than 4 h prior to the unplanned ED visit. The GP was not contacted in advance in 75.8% of the transfers, in 26.1%, because he could not be reached by phone. Most often, the final transfer decision was made by the nursing staff (52.2%).

According to the discharge letters, three quarters were admitted by ambulance (71.4%). Most common reasons for ED transfers were trauma (59.0%), which were often fall-related, urinary catheter and nutritional probe problems (overall 10.6%, male NHR 25.5%) and altered mental state (9.9%). Of the 161 letters evaluated, 156 contained diagnoses explaining the presenting chief complaint (see Table 2). Of them, 69.2% were injuries, poisoning and other consequences of external causes (S00-T98, *n* = 108), with 43.5% being head or neck injuries (S00-S19). Although injuries, poisoning and other consequences of external causes were diagnosed in seven out of 10 transfers in both sexes, categories differed. Female NHR more often had injuries of the extremities (35.1% vs. 5.9%) and those involving several body regions (14.9% vs. 5.9%). In males, 32.4% were complications due to urinary catheters (T83; 4.1% in females).

**Table 2:** Frequency of ICD-10-GM diagnoses in the ED

	Total NHR (*n* = 161)		Male NHR (*n* = 51)		Female NHR (*n* = 110)	
**Reason for transfer**^a^						
Trauma	95	59.0%	22	43.1%	73	66.4%
Altered mental state	16	9.9%	7	13.7%	9	8.2%
Infection	5	3.1%	1	2.0%	4	3.6%
Respiratory system	1	0.6%	–	–	1	0.9%
Urogenital system	5	3.1%	2	3.9%	3	2.7%
Gastrointestinal system	6	3.7%	2	3.9%	4	3.6%
Catheter and probe problems	17	10.6%	13	25.5%	4	3.6%
Cardiovascular system	6	3.7%	1	2.0%	5	4.5%
Central nervous system	1	0.6%	–	–	1	0.9%
Other	9	5.6%	3	5.9%	6	5.5%
**Diagnostic group according to ICD-10-GM**^a^						
Infectious diseases (A00-B99, J00-J22, N30, N39, N45)	7	4.5%	4	8.2%	3	2.8%
Endocrine, nutritional and metabolic diseases (E00-E99)	7	4.5%	3	6.1%	4	3.7%
Mental and behavioural disorders (F00-F99)	5	3.2%	2	4.1%	3	2.8%
Nervous system diseases (G00-G99, R40)	4	2.6%	1	2.0%	3	2.8%
Cardiovascular diseases (I00-I99, R07)	7	4.5%	1	2.0%	6	5.6%
Digestive system diseases (K00-K99, R11)	5	3.2%	1	2.0%	4	3.7%
Musculoskeletal diseases (M00-M99)	5	3.2%	–	–	5	4.7%
Injuries, poisoning and consequences of external causes	108	69.2%	34	69.4%	74	69.2%
S00-S19 Injuries of head and neck	47	43.5%	18	52.9%	29	39.2%
S20-S69 Injuries of the upper extremity	16	14.8%	2	5.9%	14	18.9%
S70-S99 Injuries of the lower extremity	12	11.1%	–	–	12	16.2%
T00-T07 Injuries involving several body regions	13	12.0%	2	5.9%	11	14.9%
T51-T65 Toxic effects of non-medically used substances	3	2.8%	–	–	3	4.1%
T79 Early trauma complications	1	0.9%	–	–	1	1.3%
T81 Complications in medical treatment	2	2.0%	1	2.9%	1	1.3%
T83 Complications due to implants in the urogenital tract	14	12.9%	11	32.4%	3	4.1%
Others (C50, N95, R04, R60, Z43)	8	5.1%	3	6.1%	5	4.7%

*ICD-10-GM* international classification of diseases, 10th version, German modification, *ED* emergency department, *NHR* nursing home resident

^a^Numbers differ due to missing values

### Extent of treatment and use of medical resources in the emergency department

Most NHR were treated by surgeons (59.0%) (see Table 3). In 93.2% (*n* = 150) of the discharge letters, information was found on diagnostic procedures performed, whereby the most frequently performed measure was physical examination (94.7%). Blood tests were performed in 26.7% (*n* = 40), with the most common requests being a small blood count (52.5%). Over half of the NHR received imaging (58.0%, *n* = 87), whereas an X-ray image was taken most often (52.0%). Other diagnostic measures such as ECG (14.0%) and urine tests (2.7%) were rarely used. 32.9% of the NHR where discharged from ED without imaging or blood test.

**Table 3:** Extent of treatment and use of medical resources in the emergency department

	Total NHR (*n* = 161)	
**Specialist department**		
Surgical	95	59.0%
Non-surgical^a^	59	36.7%
None specified	7	4.3%
**Consul of another specialist department**		
No	144	89.4%
Yes	17	10.6%
**Diagnostics**		
No	11	6.8%
Yes	150	93.2%
Blood testing	40	26.7%
ECG	21	14.0%
Urine test	4	2.7%
Physical examination	142	94.7%
Imaging (frequency *n* = 100)	87	58.0%
Computed tomography	23	23.0%
Sonography	25	25.0%
X-ray	52	52.0%
**Treatment in ED**		
Yes	137	85.1%
No	24	14.9%
No acute need for treatment	17	70.8%
**Drug Administration**		
No	94	58.4%
Yes (frequency *n* = 88)	67	41.6%
Antibiotics	13	14.8%
Anticoagulants	5	5.6%
Non-opioid Analgesics (NSAIDs)	28	31.8%
Intravenous volume substitution	15	17.0%
Sedative drugs^b^	17	19.3%
Other drugs	10	11.4%
**Trauma**^c^		
No	66	41.0%
Yes	95	59.0%
No wound care	30	31.6%
Basic wound care^d^	33	34.7%
Suturing	29	30.5%
Casting	3	3.2%
**Catheter and probe problems**		
No	144	89.4%
Yes	17	10.6%
Change of a transurethral indwelling catheter	3	17.6%
Change of a suprapubic catheter	6	35.3%
Flushing	5	29.4%
Change of a nasogastric probe	1	5.9%
No treatment	2	11.8%

*NHR* nursing home residents

^a^Including general internal medicine, neurology, ear, nose and throat, obstetrics and gynaecology, psychology, urology

^b^Including high-potent opioids, sedatives, antidepressants

^c^All cases that report a traumatic injury as the reason for transfer

^d^Including cooling, wound cleansing, dressing, tetanus vaccination, fibrin glue and wound closure strips

A total of 85.1% received treatment in the ED, 41.6% by administration of drugs. 10.6% of the discharge letters stated explicitly that there was no need for therapeutic action at the time of examination. Almost a third received non-opioid analgesics, 19.3% were given sedative drugs. Moreover, 17.0% received intravenous volume substitution.

**Fig. 1:**
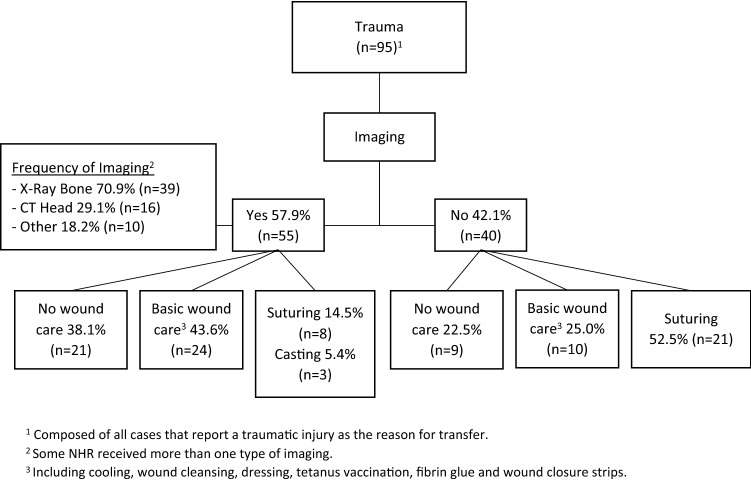
Outpatient management of nursing home residents with trauma in the emergency department

In the subgroup of NHR presenting due to trauma (*n* = 95), overall 42.1% (*n* = 40 of 95) did not receive imaging and 67.4% (*n* = 64 of 95) required basic or even no wound care (see Fig. 1). 22.1% (*n* = 21 of 95) received neither imaging nor advanced wound care. Of those receiving imaging (*n* = 55), 70.9% had an X-ray and 81.8% were discharged from the ED with basic or no wound care at all.

The presentations caused by catheter or probe related problems (*n* = 17) were either due to occlusion (41.2%) or dislocation (35.3%). These catheter-related problems were treated in 35.3% by changing an pre-existing suprapubic catheter and 29.4% by flushing the pre-existing catheter (see Table 3).

### Recommendations for further procedure and care

Regarding the further procedure (follow-up and outpatient treatment recommendation), 25.5% of the letters contained no information. Follow-up at the hospital (planned or if deteriorating) was recommended in 16.1% of NHR, in 29.8% by the GP.

72.7% of the letters contained recommendations for further outpatient treatment (*n* = 117). The most frequent suggestion was symptomatic treatment (41.8%). 19.7% of the letters stated that no further outpatient follow-up treatment was necessary.

### Quality of medical documentation

The discharge letters were on average one page long (SD 0.72), with the shortest 0.25 and the longest 3.75 pages (Median 0.75). 84.5% of the letters were machine and 15.5% were handwritten. The average completeness score was 4.18 (SD 0.99), although only 50.3% of the discharge letters achieved a score of 5, thereby meeting the minimum requirements of the VHitG.

## Discussion

In this study, most of the unplanned ED transfers happened during working hours, but the GP was rarely contacted in advance. The most common reasons for transfer were trauma, most often resulting in injuries of the head or neck, and catheter or probe problems. Injuries were often reasons in female and catheter-related problems in male NHR. Our study showed that two thirds of injured NHR discharged from the ED required no or only basic wound care beforehand. Overall, almost a third of the NHR where discharged from the ED without imaging or blood test. The most frequent suggestion for further outpatient treatment was symptomatic and follow-up by the GP.

Consistent with current research [5, 8, 19], the most common reason for unplanned outpatient ED visits was fall-related trauma. Compared to peers living at home, the incidence of falls is two to three times higher among NHR [20]. However, it is estimated that only one in ten falls causes a serious injury requiring hospital treatment [21]. Our study results show that in fact only one third of injured NHR require advanced wound care in the ED, with the majority of these treated by suturing, which would also be possible in an outpatient setting by GPs. A third was discharged from ED without any wound care at all. This finding is also confirmed by a study by Burke et al. [11], calling the need for such visits into question. However, assessing the need for further evaluation after falls is particularly difficult, because less than 30% of NHR can provide an accurate summary of the accident sequence and struggle to describe their symptoms clearly [22]. Our data suggest that injuries of the head and neck were over 40% of the fall consequences. Even if there are no obvious injuries to the head, involvement can often not be ruled out with certainty, especially because elderly show atypical neurological findings in the case of intracranial haemorrhages and increased intracranial pressure [23]. The Glasgow Coma Scale (GCS) often underestimates the extent of the damage [24]. Therefore, some authors call for older patients to always undergo a CT scan after a fall—even if haemodynamics, level of consciousness and GCS value are normal [25, 26]. This leads to radiological overdiagnosis, often without consequences, as it is estimated that in elderly patients less than 5% develop CT findings requiring neurosurgical intervention after a fall on the head [26]. Established diagnostic tests can reduce the number of unnecessary CT scans [27], yet they are often only applicable to NHR to a limited extent. The underlying reasons are pre-existing abnormal neurological findings for example due to existing dementia or increased bleeding risk due to therapy with oral anticoagulants. So far, there are no concrete guidelines in Germany on how to deal with elderly fall patients. In its guideline, the German College of General Practitioners and Family Physicians (DEGAM) recommends ruling out injuries after falls with ‘sufficient certainty’ and treating them ‘adequately’ [28]. However, in our study, only 25% of the NHR received a CT of the head after a fall. As treatment in ED usually does not exceed care by a GP, NHR with traumatic injuries should primarily be seen by a GP. Overall, these results highlight the need for establishing algorithms or guidelines especially for elderly patients to support a targeted differentiation of fall severity and the associated assessment of the need for treatment in the ED. Identifying the group of NHR who do not need further diagnostics already in the NH, could avoid many transfers.

The second most common transfer reason was problems with catheters, which are responsible for over a quarter of all ED visits in the group of male NHR. Over half of these catheter-related visits were due to occlusion and were mostly treated by changing an existing catheter or flushing the pre-existing one. NHR are often catheterized for weeks to months or longer, while the indications for insertion of a urinary catheter are wide-ranging [29]. Within Europe, the proportion of NHR with indwelling urinary catheters (IUCs) varies from 0 to 23%, with significantly more male than female NHR. In Germany, it is estimated that over 15% of male NHR are supplied with IUC, which is the second highest rate in Europe [30]. Men are more likely to have suprapubic catheter and women more likely to have transurethral catheter [29–31]. In most cases, women’s IUCs are changed by the nursing staff on site, while men—regardless of whether suprapubic or transurethral—are not [32]. In many NHs there is an internal policy that catheters are not placed or changed in men, although there is no stipulation for this. On the contrary, the National Association of Statutory Health Insurance Physicians even demands that the nursing facility must ensure the change, insertion or removal of a transurethral IUC as well as the care of a suprapubic catheter [33]. When NHR are taken to hospital for changing or flushing an IUC, usually by ambulance, it is not only burdensome for the NHR, but also incurs high costs. Better coordination and consultation with GPs, as well as improved training of nurses in the management transurethral and suprapubic IUCs could reduce the number of ED visits, especially for male NHR.

### Limitations and strengths

This is one of the first studies combing information from questionnaires and discharge letters regarding unplanned ED visits by NHR, which did not lead to hospitalization. The data examined were taken from a convenience sample of 14 NHs in northwestern Germany. Therefore, selection bias, which can affect the generalizability of all German NHs, cannot be exempted. However, regarding facility sizes, regions (urban and rural) and sponsorships (non-profit and private-for-profit), we tried to include a heterogeneous sample of NHs. Beyond that, no structural data on the organisation of NHs, such as composition and qualification of NH staff, was available. It is possible that personnel differences influenced the transfer decision.

Furthermore, it is possible that not all transfers carried out during study period were reported by NH staff (under-reporting). A social desirability bias can arise when estimating 6-month mortality, i.e., nursing staff may present themselves or their facility more favourably. However, questionnaires were evaluated completely anonymously to reduce this kind of response. Even though most of the information regarding the NHR characteristics should have been assessed by NH staff relying on existing medical records, a further possible limitation is a recall bias.

About the evaluation of the discharge letters, a limitation is that they were only coded by one person. Furthermore, it is possible that more medical procedures were carried out than subsequently documented in the discharge letters. This is especially true for blood tests, as the corresponding laboratory results were sometimes sent incompletely or possibly not at all.

To assess this possible impact, we evaluated the completeness of the letters using a score.

## Conclusions

Falls and IUCs are main causes for unplanned ED visits. Our data suggest that the diagnostic and therapeutic interventions performed in the ED, often do not exceed GP care and many ED visits might, therefore, be unnecessary. Our results indicate that both GPs and EDs are not adequately fulfilling their mandate to provide care to NHR. A large proportion of NHR were send back with no examination or very limited one and with discharge letter not including all basic information. In addition, better training of nurses in the use of IUCs and a revision of internal algorithms in NHs on how to deal with falls and IUCs, could avoid outpatient ED transfers, especially for male NHR. The reinsertion of a transurethral catheter or even the flushing of a suprapubic catheter could be done safely by qualified nursing staff. However, there is a need for more studies looking at IUC use and its management in NHR.

## Data Availability

The datasets supporting the conclusions of this article are available from the corresponding author on reasonable request.
